# Remarks by Dr. R. Coates

**Published:** 1842-08-13

**Authors:** 


*Remarks.*—Sir Henry is inclined to attribute the occurrence of these rare and singular phenomena to a change in actually living parts, reducing them to a condition "perfectly analogous to the first and earliest stage of decomposition," the light being "evolved precisely in the same manner as this phenomenon is produced in the dissecting room, in burial grounds, and in marine animals during the early stage of decay." There is no doubt that many alterations somewhat analogous *in appearance* to chemical decompositions, are continually going on during life—the changes now well known to occur in the globules of the blood during inflammation, and other diseases, are a sufficient proof of this fact—but the value of conclusions drawn from such predicates is, in the present state of science, exceedingly doubtful. If the old opinion, that vital action is the antagonist and not the result of a mere modification of chemical law, (a doctrine the foundations of which, it must be confessed, are beginning to be threatened pretty loudly,) is to be overthrown, it must be by much stronger evidence than that presented by the evidence of such cases as those above recorded. We can perceive nothing in the phenomena noticed, other than the proof of morbid or misplaced secretions in some conditions of the constitution, in animals which do not present them at all, or present them only in other places during health. There are some plants which, at certain hours, envelope themselves in a halitus of inflammable gas sufficient in quantity to yield a flash over their stem and branches on the touch of a candle, and even their leaves have been injured by the heat, when the experiment has been too often tried. Why, then, may not disease occasion similar eliminations from the animal frame? As to the secretion of *light*, even during health, it is so common a property of animal life, that it would be almost as reasonable to wonder at its habitual absence in the more noble animals, than to be surprised at its occasional presence in them from morbid causes. Whether the phenomena under notice are regarded as the result of combustion or simple luminous secretion, they certainly do not transcend the analogies of living nature, and whether their action be interstitial, as in the latter cases, or superficial in the shape of exhalations, as in the former, they are attributable immediately to the agency of matters which may be properly regarded, we think, as vital products foreign to the living parts, and in their nature excrementitious.

